# Infant feeding among HIV-positive mothers and the general population mothers: comparison of two cross-sectional surveys in Eastern Uganda

**DOI:** 10.1186/1471-2458-9-124

**Published:** 2009-05-07

**Authors:** Lars T Fadnes, Ingunn MS Engebretsen, Henry Wamani, Nulu B Semiyaga, Thorkild Tylleskär, James K Tumwine

**Affiliations:** 1 Centre for International Health, University of Bergen, Bergen, Norway; 2 Makerere University School of Public Health, Kampala, Uganda; 3 Department of Paediatrics and Child Health, Makerere University, Makerere, Uganda

## Abstract

**Background:**

Infant feeding recommendations for HIV-positive mothers differ from recommendations to mothers of unknown HIV-status. The aim of this study was to compare feeding practices, including breastfeeding, between infants and young children of HIV-positive mothers and infants of mothers in the general population of Uganda.

**Methods:**

This study compares two cross-sectional surveys conducted in the end of 2003 and the beginning of 2005 in Eastern Uganda using analogous questionnaires. The first survey consisted of 727 randomly selected general-population mother-infant pairs with unknown HIV status. The second included 235 HIV-positive mothers affiliated to The Aids Support Organisation, TASO. In this article we compare early feeding practices, breastfeeding duration, feeding patterns with dietary information and socio-economic differences in the two groups of mothers.

**Results:**

Pre-lacteal feeding was given to 150 (64%) infants of the HIV-positive mothers and 414 (57%) infants of general-population mothers. Exclusive breastfeeding of infants under the age of 6 months was more common in the general population than among the HIV-positive mothers (186 [45%] vs. 9 [24%] respectively according to 24-hour recall). Mixed feeding was the most common practice in both groups of mothers. Solid foods were introduced to more than half of the infants under 6 months old among the HIV-positive mothers and a quarter of the infants in the general population. Among the HIV-positive mothers with infants below 12 months of age, 24 of 90 (27%) had stopped breastfeeding, in contrast to 9 of 727 (1%) in the general population. The HIV-positive mothers were poorer and had less education than the general-population mothers.

**Conclusion:**

In many respects, HIV-positive mothers fed their infants less favourably than mothers in the general population, with potentially detrimental effects on both the child's nutrition and the risk of HIV transmission. Mixed feeding and pre-lacteal feeding were widespread. Breastfeeding duration was shorter among HIV-positive mothers. Higher educational level and being socio-economically better off were associated with more beneficial infant feeding practices.

## Background

For mothers in Sub-Saharan Africa, an appropriate choice of infant feeding is fundamental to optimising infant survival and minimising infant morbidity. Promotion of exclusive breastfeeding has the potential to prevent 8% of child mortality, or save 37 million disability-adjusted life years every year [1,2]. It is well documented that exclusive breastfeeding can benefit infants of HIV-negative mothers [1,3-5]. How to optimise survival and avoid morbidity among the infants and children of HIV-positive mothers is an ongoing discussion [6-10]. Replacement feeding can reduce HIV-transmission, but is also associated with morbidity related to diarrhoea and respiratory infections [11,12]. For mothers without access to piped water and cooking fuel, or who have not disclosed their HIV-status, replacement feeding does not seem to increase HIV-free survival [13].

Practical implementation of the previous infant feeding recommendations for HIV-positive mothers from the World Health Organisation (WHO) has often created confusing messages resulting in disadvantageous feeding patterns, mixed feeding in particular [14-16]. Compared to exclusive breastfeeding, mixed feeding is associated with increased morbidity and mortality for infants of both HIV-positive and HIV-negative mothers, and with increased HIV transmission for HIV-positive mothers [2-5,8,9,17]. A concern is that promotion of replacement feeding to infants of HIV-positive mothers has created a spill-over effect among the infants of HIV-negative mothers, resulting in increased usage of maternal milk replacements including formula milk [16,18].

The aim of this study was to compare feeding practices, including breastfeeding, between infants and children under the age of two years born to HIV-positive mothers and infants born to general-population mothers in Eastern Uganda.

## Methods

### Study settings

**Figure 1:**
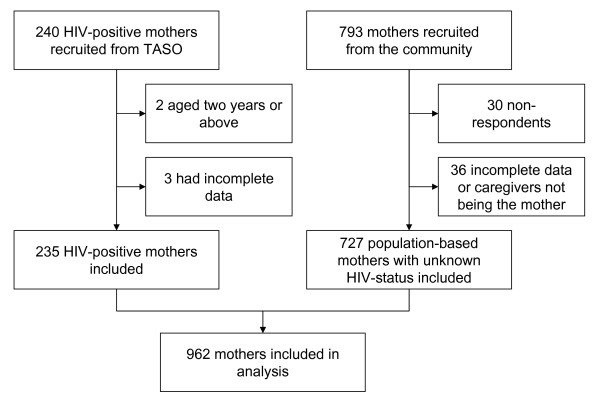
**Study enrolment overview**.

This study compares two cross-sectional surveys conducted in the end of 2003 and the beginning of 2005 in the same area in Eastern Uganda using analogous questionnaires (Figure 1).

Mbale district has a population of 720,000, predominantly Bagisu people, with 90% living in rural areas and being subsistence farmers. The overall literacy rate is 64% for men and 49% for women [19]. Uganda has an estimated national HIV-prevalence of 7.5% in women aged 15 – 49 years (2005) [20].

The first survey was a community-based study conducted in 2003 and included 793 randomly-selected caretaker-infant pairs from urban and rural areas in Mbale district [21]. The infants were 0 – 11 months old. Owing to non-responses (n = 30) and incomplete data (n = 36), 727 mother-infant pairs remained in the analysis. The recruitment and selection procedures have been reported previously [21]. We did not collect information about the mothers' HIV status. These participants are referred to as "general-population mothers."

The second survey was conducted in 2005 and included 240 mother-child pairs. This study was performed in collaboration with The Aids Support Organisation (TASO) [22]. TASO is a non-governmental organisation working for HIV-positive people in Uganda. It provides counselling, information, support and medical treatment for HIV-positive people. The mothers were approached through TASO-Mbale, including their outreach clinics in Mbale district and adjacent areas. The children were 0 – 23 months old. Comparisons of infants were made with corresponding age-groups in the general population. Consecutive sampling was used to recruit these participants. All the mothers approached agreed to participate in the study. Five mother-infant pairs were excluded from analysis owing to missing information or to the child being at least 24 months old. All women recruited through TASO were known to have HIV-positive status and are described as HIV-positive mothers in this study.

Data from both surveys were merged yielding a total of 962 mothers-infants pairs for analysis. There were no case overlaps between the two cross-sectional studies.

To increase reliability, a total of 20 mothers were re-interviewed by different data collectors some days or weeks after the initial interview. The agreement between the initial interviews and the reliability interviews was generally high.

### Data management

The structured interviews in both surveys were based on analogous questionnaires. It contained topics concerning infant feeding practices including breastfeeding, feeding knowledge, mother's and father's education, occupation and household assets. We examined a list of thirty liquid, semi-solid and solid foods using 24-hour dietary recall. The questionnaire for the HIV-positive mothers also contained questions regarding time of HIV diagnosis and participation in the PMTCT-program. Time of HIV-diagnosis was used to categorize mothers who got their HIV-diagnosis before birth and mothers who got their diagnosis after birth. We pre-tested the questionnaires and worked with data collectors who were fluent in the local language, Lumasaba, and English to conduct the interviews. Data were entered in EpiData 3.1 and SPSS 14 was used for data analysis.

### Definitions

Feeding information was based on WHO definitions and recommendations [23], as follows. Exclusive breastfeeding: giving breast milk only, except for medicines and vitamin or mineral supplements; predominant breastfeeding: breast milk is nutritionally dominant, but with the possible addition of water-based fluids, fruit juices, tea without milk or oral rehydration salts; mixed feeding: non-human milk, semi-solids or other solids given in addition to breast milk; replacement feeding: breastfeeding stopped or never being given any breast milk. Exclusive replacement feeding was defined as never having given any breast milk. Prelacteal feeding was defined as any food item or liquid other than breast milk given to the infant during the first 3 days after delivery.

### Statistics

Baseline characteristics were examined utilising frequency tables and cross-tabulations with Pearson *χ*^2^. One-way analysis of variance (ANOVA) was used to calculate factors influencing the nutritional items given to the children and a linear regression model was used to investigate associations between education, socio-economic status and HIV-status, controlling for living area. Breastfeeding duration was analysed by Kaplan-Meier survival statistics.

All the mothers in the merged data were grouped socio-economically into quintiles on the basis of wealth assessment, using principal component factor analysis [24]. Housing characteristics and assets including toilet facilities, number of rooms and beds, roof material, lantern, radio, television, bicycle and motor vehicles were included in the model. Quintiles were inferred from the first principal component. This method is recognised as a good proxy for household wealth [25]. The Mann-Whitney-Wilcoxon test for independent samples was used to compare socio-economic ranks.

The results will be presented in the following order: First, we will present data from all mother-infant-pairs with pre-lacteal feeding and breastfeeding duration as the main topics. Second, we will include only mothers with infants under one year old, focusing on feeding patterns based on 24-hour recall. Third, we will examine breastfeeding initiation time, comparing general-population with HIV-positive mothers, the latter being stratified into those who were diagnosed pre-natally and those who were diagnosed post-natally. Lastly, we will present the differences in socio-economic status.

### Ethics

Ethical approvals were granted from Makerere University, Faculty of Medicine Ethics and Research Committee, the Uganda National Council for Science and Technology and the Regional Committee for Medical Research Ethics, Western Norway. Informed consent was obtained from each mother prior to study participation.

## Results

The HIV-positive mothers were older than the general-population mothers, median age 30 years (inter-quartile range 28 – 35) versus 24 years (IQR 20 – 30) (Table 1). The general-population mothers were more educated than the HIV-positive mothers. There was no difference in the education levels of the fathers of the infants in the two groups. Farming was the dominant occupation among both groups of mothers, but the general-population cross-sectional survey included more urban mothers than the TASO-affiliated survey among HIV-positive mothers. Marital status was dissimilar in the two groups. Most of the general-population mothers reported being married or cohabiting, with 19 (3%) being widowed, separated or divorced. In contrast, only 90 (39%) of the HIV-positive mothers were married, half were widowed and some were separated or divorced. HIV-positive mothers had more children and lived under more crowded conditions than their counterparts in the general population. Socio-economically, the HIV-positive mothers were more often among the poorest and less often among the least poor.

**Table 1:** Baseline characteristics for HIV-positive mothers and mothers from the general population

	HIV-positive n = 235 (%)	General-population mothers n = 727 (%)	Chi-square *p (χ^2^)*
**Gender of infant**			
Girl	109 (46)	346 (48)	
Boy	126 (54)	381 (52)	
			
**Age of infant**			
< 6 months	37 (16)	415 (57)	
6 – 11 months	53 (23)	312 (43)	
12 – 17 months	64 (27)		
18 – 23 months	81 (34)		
			
**Mother's education**			
None	27 (11)	59 (8)	*< 0.01*
Stopped in primary	125 (53)	301 (41)	
Completed primary (7 years)	39 (17)	139 (19)	
Secondary education	37 (16)	183 (25)	
Higher education (12 years and above)	7 (3)	45 (6)	
			
**Father's education**			
None	14 (6)	40 (6)	
Stopped in primary	72 (32)	161 (26)	
Completed primary (7 years)	62 (27)	154 (25)	
Secondary education	50 (22)	177 (29)	
Higher education (12 years and above)	30 (13)	83 (14)	
			
**Mother is farming**			
Yes	201 (86)	507 (70)	*< 0.001*
No	34 (14)	218 (30)	
			
**Marital status**			
Married or cohabiting	91 (39)	667 (92)	*< 0.001*
Widowed	112 (48)	6 (1)	
Separated or divorced	28 (12)	13 (2)	
Single	4 (2)	41 (6)	
			
**Owning land and/or house**			*< 0.01*
Yes	162 (69)	561 (78)	
No	73 (31)	154 (22)	
			
Rural	205 (87)	401 (55)	*< 0.001*
Urban	30 (13)	326 (45)	
			
**Socio-economic wealth index**			
Poorest quintile	65 (28)	122 (17)	*< 0.001*
2^nd ^quintile	49 (21)	141 (20)	
3^rd ^quintile	52 (22)	143 (20)	
4^th ^quintile	38 (16)	148 (21)	
Least poor quintile	30 (13)	159 (22)	
			
**Mother's age**			*< 0.001*
≤ 19	2 (1)	129 (18)	
20 – 24	20 (9)	247 (34)	
25 – 29	61 (26)	146 (20)	
30 – 34	85 (36)	134 (19)	
≥ 35	67 (29)	64 (9)	
			
**Number of siblings**			*< 0.001*
None	12 (5)	175 (24)	
1	41 (17)	148 (20)	
2–3	82 (35)	196 (27)	
≥ 4	100 (43)	205 (28)	
			
**Crowdedness, no. of people per room**			*< 0.001*
0 – 2	43 (18)	155 (21)	
2 – 4	68 (29)	343 (47)	
4 – 6	67 (29)	142 (20)	
≥ 6	57 (24)	86 (12)	

### Early feeding practices

**Figure 2:**
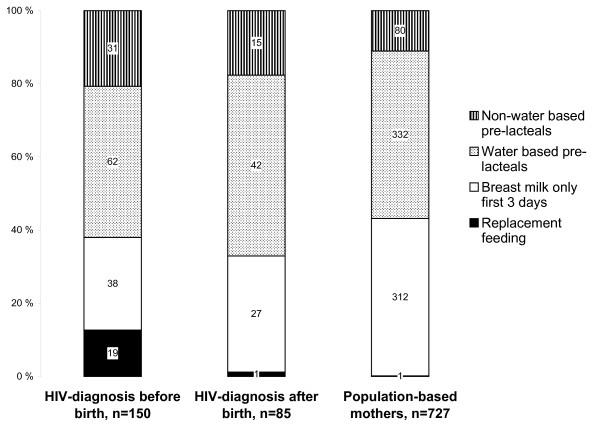
**Initial feeding practices during the first 3 days comparing HIV-positive mothers diagnosed before and after birth and mothers from the general population**. ^1 ^Exclusive replacement feeding significantly different between HIV-positive diagnosed before birth and general-population mothers (p < 0.001). ^2 ^Non-water based pre-lacteals were given significantly more often to children of HIV-positive mothers than general-population mothers.

**Figure 3:**
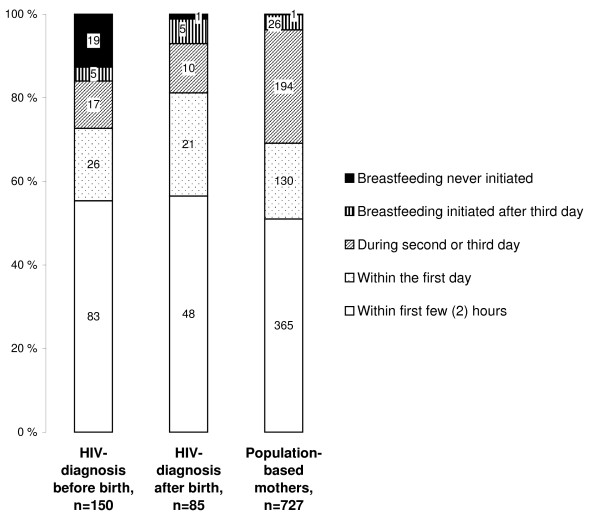
**Breastfeeding initiation time comparing HIV-positive mothers diagnosed before and after birth and mothers from the general population**. ^1 ^Exclusive replacement feeding significantly different between HIV-positive mothers acquiring HIV prior to birth and general-population mothers (p < 0.001).

More than half the mothers gave something in addition to breast milk during the first three days (Figure 2). Pre-lacteal feeding was more often non-water-based, including non-human milk, among the HIV-positive mothers than among the general-population mothers. Breastfeeding was initiated within the first few hours by approximately half the mothers and within the first day by three-quarters in both groups (Figure 3). Pre-lacteals were less commonly given by more educated mothers (Table 2). HIV-positive mothers with many children gave pre-lacteal feeding more often than mothers with few children. Mother's age, marital status and owning house or land were not significantly associated with the initial feeding patterns in either group. Among the general-population mothers, feeding breast milk only during the first three days was associated with the father having higher education and the mother not being farmer, whilst these variables were not significantly associated with initial exclusive breastfeeding among the HIV-positive mothers. The better-off among the general-population mothers were exclusively breastfeeding more often than the poorer mothers during the first three days, while the HIV-positive mothers who were better-off opted for exclusive replacement feeding more often than their poorer peers. Education of the mothers was also associated with a higher rate of exclusive replacement feeding among the HIV-positive mothers and a higher proportion of initial exclusive breastfeeding among the general-population mothers.

**Table 2:** Feeding patterns during the first 3 days among HIV-positive mothers compared to mothers from the general population

	HIV-positive n = 235 (%).			General-population mothers n = 727 (%)	
	Pre-lacteals given	Breast milk only first 3 days	Exclusive replacement feeding	Pre-lacteals given	Breast milk only first 3 days
**Gender of infant**					
Girl	68 (62)	31 (28)	10 (9)	200 (58)	146 (42)
Boy	82 (65)	34 (27)	10 (8)	214 (66)	166 (44)
					
**Mother's education**		*		*	
None	14 (52)	13 (48)	0 (0)	36 (61)	23 (39)
Stopped in primary	87 (70)	31 (25)	7 (6)	187 (62)	114 (38)
Completed primary (7 years)	25 (64)	8 (21)	6 (15)	84 (60)	54 (39)
Secondary education	21 (57)	10 (27)	6 (16)	86 (47)	97 (53)
Higher education (12 years and above)	3 (43)	3 (43)	1 (14)	21 (47)	24 (53)
					
**Father's education**				**	
None	8 (57)	6 (43)	0 (0)	27 (68)	13 (32)
Stopped in primary	44 (61)	25 (35)	3 (4)	112 (70)	48 (30)
Completed primary (7 years)	43 (69)	13 (21)	6 (10)	94 (61)	60 (39)
Secondary education	31 (62)	14 (28)	5 (10)	89 (50)	88 (50)
Higher education (12 years and above)	19 (63)	7 (23)	4 (13)	33 (40)	50 (60)
					
**Mother is farming**				**	
Yes	131 (65)	56 (28)	14 (7)	308 (61)	199 (39)
No	19 (56)	9 (26)	6 (18)	105 (48)	112 (51)
					
**Marital status**					
Married or cohabiting	61 (67)	23 (25)	7 (8)	379 (57)	287 (43)
Widowed	71 (63)	32 (29)	9 (8)	4 (67)	2 (33)
Separated or divorced	16 (57)	9 (32)	3 (11)	10 (77)	3 (23)
Single	2 (50)	1 (25)	1 (25)	21 (51)	20 (49)
					
**Owning land and/or house**					
Yes	100 (62)	51 (31)	11 (7)	331 (59)	229 (41)
No	50 (69)	14 (19)	9 (12)	77 (50)	77 (50)
					
Rural	132 (64)	57 (28)	16 (8)	239 (60)	162 (40)
Urban	18 (60)	8 (27)	4 (13)	175 (54)	150 (46)
					
**Socio-economic wealth index**		*		**	
Bottom quintile	39 (60)	23 (35)	3 (5)	74 (61)	48 (39)
2^nd ^quintile	39 (80)	8 (16)	2 (4)	92 (65)	49 (35)
3^rd ^quintile	33 (63)	16 (31)	3 (6)	80 (56)	63 (44)
4^th ^quintile	22 (58)	10 (26)	6 (16)	84 (57)	64 (43)
Top quintile	17 (57)	7 (23)	6 (20)	76 (48)	82 (52)
					
**Mother's age**					
≤ 19	2 (100)	0 (0)	0 (0)	76 (59)	53 (41)
20 – 24	13 (65)	6 (30)	1 (5)	136 (55)	111 (45)
25 – 29	38 (62)	16 (26)	7 (11)	87 (60)	59 (40)
30 – 34	54 (64)	24 (28)	7 (8)	74 (55)	60 (45)
≥ 35	43 (64)	19 (28)	5 (7)	35 (55)	28 (44)
					
**Number of children**		**			
One	6 (50)	6 (50)	0 (0)	98 (56)	77 (44)
2	24 (59)	7 (17)	10 (24)	79 (53)	69 (47)
3–4	53 (65)	22 (27)	7 (9)	117 (60)	78 (40)
≥ 5	67 (67)	30 (30)	3 (3)	117 (57)	88 (43)
					
**Crowdedness, no. of people per room**		*			
0 – 2	26 (60)	8 (19)	9 (21)	87 (56)	68 (44)
2 – 4	45 (66)	18 (27)	5 (7)	194 (57)	148 (43)
4 – 6	45 (67)	18 (27)	4 (6)	83 (58)	59 (42)
≥ 6	34 (60)	21 (37)	2 (4)	50 (58)	36 (42)
					
**HIV-diagnosis related to infant birth**		**			
HIV-diagnosis before birth	93 (62)	38 (25)	19 (13)		
HIV-diagnosis after birth	57 (67)	27 (32)	1 (1)		

Exclusive replacement feeding column is removed from the general population because it was chosen by only one mother from this group

* Significance level of p < 0.05, ** Significance level of p < 0.01

### Breastfeeding duration

**Figure 4:**
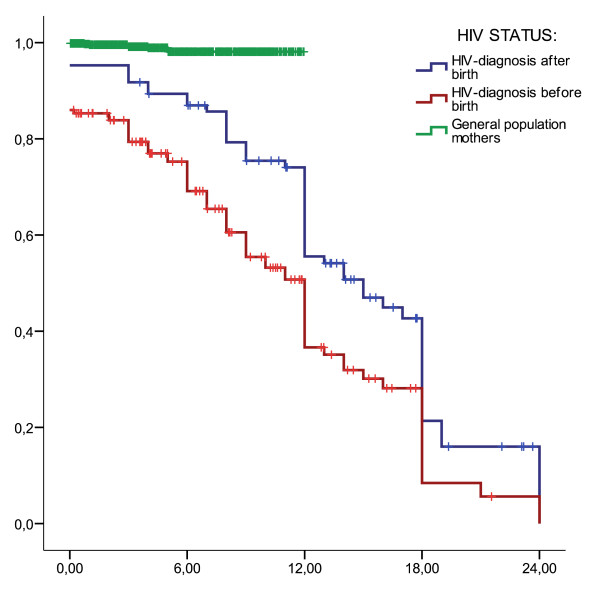
**Breastfeeding duration in months (x-axis) stratified for HIV-positive mothers diagnosed before and after delivery and general-population mothers**. Proportion still breastfeeding (y-axis) visualised with a Kaplan-Meier-plot.

The median breastfeeding duration among the HIV-positive mothers was 12 months (95% confidence interval 11.5 to 12.5) (Figure 4). In the group of general-population mothers with infants under one year old, only 9 out of 727 (1%) mothers had stopped breastfeeding. In contrast, 24 out of 90 (27%) HIV-positive mothers with infants under 12 months old had stopped breastfeeding. Breastfeeding duration was significantly shorter among the HIV-positive mothers than among the general-population mothers (Mantel-Cox log rank test p < 0.001). There was a shorter breastfeeding duration among HIV-positive mothers who were diagnosed before birth, with a median of 12 months (95% C.I. 10.4 – 13.6), in contrast to 15 months (95% C.I. 10.5 – 19.5) among those diagnosed post-partum (Log Rank test: p < 0.05).

### Breastfeeding patterns and dietary information

**Figure 5:**
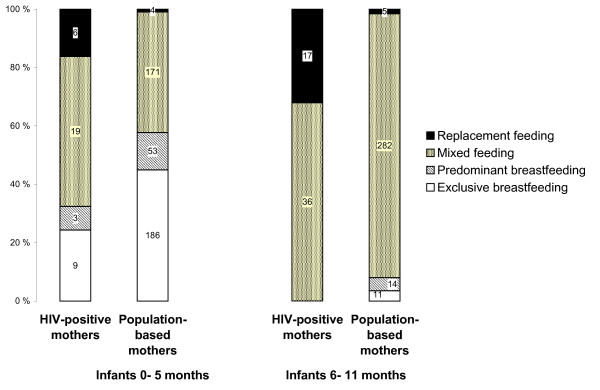
**Age-specific infant feeding patterns for infants of HIV-positive mothers and general-population mothers according to 24-hour recall**. ^1 ^Infants aged 0–5 months: general-population mothers practised exclusive breastfeeding more frequently than HIV-positive mothers (p < 0.05), while the opposite was seen with replacement feeding (p < 0.001). Among infants aged 6–11 months the difference in frequency was different (p < 0.001).

Exclusive replacement feeding was reported by 20 (8.5%) HIV-positive mothers and one (0.1%) mother from the general population. Among the 20 HIV-positive mothers practising exclusive replacement feeding, all except one were diagnosed HIV-positive prior to birth. Based on the 24-hour dietary recall, half the general-population mothers exclusively breastfed their infants under 6 months old, in contrast to a quarter of the HIV-positive mothers (Figure 5). Approximately half the mothers in both groups gave their infants mixed feeding. Half the infants below 6 months of age born to HIV-positive mothers received water, non-human milk and staple food including bananas, maize and beans (Table 3). Fewer mothers from the general population gave these food items to their infants under 6 months old. HIV-positive mothers gave more food items to their infants than the mothers in the general population.

**Table 3:** Nutritional items given to infants of HIV-positive mothers and mothers in the general population using 24-hour recall (age categorised)

**Food items (age categorised)**	HIV-positive n = 235 (%)	General-population mothers n = 727 (%)	Chi-square *p (χ^2^)*
**0 – 5 months**			
Water	20 (54)	94 (23)	*< 0.001*
Herbal water or gripe water	5 (17)	24 (6)	
Fruit juice, tea with sugar etc	10 (27)	68 (16)	
Non-human milks	20 (54)	146 (35)	*< 0.05*
Staple food (maize, beans etc)	18 (49)	60 (14)	*< 0.001*
Meat, egg or fish	2 (5)	8 (2)	
			
Number of items given last 24 hours			*< 0.001*
None	9 (24)	187 (45)	
1 – 2	11 (30)	152 (37)	
3 – 5	8 (22)	63 (15)	
6 – 9	5 (14)	11 (3)	
10 or more	4 (11)	1 (0.2)	
			
**6 – 11 months**			
Water	43 (84)	220 (71)	*< 0.05*
Herbal water or gripe water	2 (4)	16 (5)	
Fruit juice, tea with sugar etc	26 (51)	142 (46)	
Non-human milks	33 (65)	171 (55)	
Staple food (maize, beans etc)	50 (98)	252 (81)	*0.001*
Meat, egg or fish	20 (39)	55 (18)	*0.001*
			
Number of items given last 24 hours			*0.001*
None	0 (0)	11 (4)	
1 – 2	2 (4)	75 (24)	
3 – 5	19 (37)	121 (39)	
6 – 9	24 (47)	80 (26)	
10 or more	6 (12)	25 (8)	

Infants over 6 months old were mostly mixed fed with a third of the HIV-positive mothers practising replacement feeding. Some of the general-population mothers with infants over 6 months old still gave only breast milk and clear liquids. Staple foods were universally given by the HIV-positive mothers and by most of the mothers in the general population. Protein-containing items such as meat, fish and eggs were given to almost half the infants over 6 months old of HIV-positive mothers, but to less than a fifth of the infants of general-population mothers.

Among infants less than 1 year of age, 3 (3%) belonging to HIV-positive mothers and 12 (2%) to general-population mothers had been given local brew or other alcohol containing liquids since birth. The alcohol content of the local brew is usually considerably lower than in e.g. beer. In children of HIV-positive mothers aged 12 – 23 months, 28 (19%) had ever received alcoholic liquids as local brew.

### Socio-economic differences

The HIV-positive mothers were poorer than the mothers from the general population. When the first factor from the principal component analysis of socio-economic status was ranked, HIV-positive had a mean rank of 399 in contrast to 498 in the general population (lowest rank indicating the poorest, Mann-Whitney-Wilcoxon Z = -4.8, p < 0.001). Using a linear regression model, both living area and HIV status were independently associated with socio-economic wealth (R^2 ^= 0.08, p < 0.05 for the model and each of the factors). Similarly, mother's education was associated with both living area and HIV-positive status, though the effect was small (R^2 ^= 0.05, p < 0.05 for the model and each factor independently). There were some regional differences in socio-economic status. Among the study participants from the areas in Kumi, 31 out of 46 (67%) belonged to the poorest quintile.

The mean number of food items given to infants aged 6 – 11 months based on 24-hour recall ranged from 3.9 (95% C.I. 3.3 – 4.5) among the poorest socio-economic quintile to 5.8 (95% C.I. 5.0 – 6.6) in the least poor group (F(4, 349) = 4.5, p < 0.001; the poorest group received significantly fewer food items than the two least poor groups). The difference was less pronounced for infants above 12 months and for those under 6 months old.

## Discussion

In contrasting these surveys of infant feeding practices among HIV-positive mothers on the one hand and the general-population mothers on the other, a number of issues arise. The first and most worrying is the fact that in several aspects of infant feeding the HIV-positive mothers seem to choose the least good option more frequently than the general population. Among the infants below 6 months of age, HIV-positive mothers chose mixed breastfeeding more often than the general population, and they were less likely to breastfeed their infants exclusively. We know from earlier studies that mixed breastfeeding is the least safe infant feeding practice for children born to HIV-positive mothers [2-5,8,9,17]. In addition, half the HIV-positive mothers had introduced staple food to their infants below the age of six months compared to a quarter of the population-based mothers. Early introduction of solid foods combined with breastfeeding has been shown to increase the risk of vertical HIV transmission four-fold [10].

Second, pre-lacteal feeding was practised by most mothers in both groups. Our prevalence is higher than was reported from Western Uganda, where 43% gave pre-lacteal feeds to their infants [26]. Pre-lacteal feeding has been associated with increased risk for neonatal deaths [27]. Among the HIV-positive mothers, pre-lacteal feeding more often included non-human milk. Maybe more of these mothers initially considered exclusive replacement feeding? Exclusive replacement feeding was ultimately chosen by some HIV-positive mothers who were diagnosed prior to birth, but hardly at all among other mothers.

On the other hand, infants over 6 months old born to HIV-positive mothers received a varied diet more often than their peers from the general population. HIV-positive mothers might have made extra efforts to give their infants good and varied diets in spite of a challenging socio-economic situation. Our impression was that nutrition was emphasised during the counselling sessions for HIV-positive mothers. The least poor mothers also gave more food items to their infants than the poorest. This may suggest that wealth influenced infant feeding. In terms of avoiding mixed feeding, mothers who were more educated or socio-economically better-off fed their infants more beneficially than their less educated and poorer peers. Similar findings associating infant feeding with education and wealth have also been described in studies from both Eastern- and Western Uganda [26,28,29]. Half the HIV-positive mothers were widowed, which may in part explain why education of the fathers of these infants was less associated with feeding practices than was the case in the general population.

Some of the infants over 6 months old in the general population received nothing except breast milk and clear fluids, which WHO considers to be inadequate complementary feeding [30]. This was not seen among the infants of HIV-positive mothers. Inadequate complementary feeding at the age of 6 months has been shown to be a predictor for impaired growth and stunting up to at least the age of 18 months [31].

Breastfeeding duration was clearly shorter among the HIV-positive mothers, with a median duration of 12 months. The survey of the general-population mothers was not designed to evaluate breastfeeding duration, but according to the demographic health survey in Uganda the median breastfeeding duration was 19.9 months [32]. Mothers who were diagnosed HIV-positive prior to delivery breastfed their infants for a shorter time than those who were diagnosed later. We cannot measure whether this reduction in breastfeeding duration was beneficial; some reports have suggested that promoting early weaning does not reduce HIV-free survival [7].

The time of initiation of breastfeeding was similar for HIV-positive and general-population mothers except for the proportion opting to exclusively replacement-feed. In both groups, breastfeeding was commonly introduced with a delay of more than a few hours after birth. Delayed breastfeeding initiation has been reported to increase the risk of neonatal death [17]. Encouraging earlier breastfeeding initiation could thus increase survival in this setting.

There might be many reasons for these observed differences. First, the risk of transmission through breast milk might have been stressed to many HIV-positive mothers during counselling, so they might have wanted to reduce the extent of breastfeeding. Some mothers might have wanted to practise exclusive replacement feeding, but failed to do so because of social pressure, economic reasons or lack of access to formula which has been described in many different settings, including South Africa [15]. Further, these women might have been less empowered and used primary health care facilities less than women in the general population, thereby losing some of the preventive health messages provided at antenatal care units, including promotion of exclusive breastfeeding. Differences in intervention-coverage between different socio-economic groups have been reported in other studies [33], but we found no significant association between socio-economic status and attending the PMTCT program.

We know from interviewing the health staff, including counsellors in TASO, that most of the HIV-positive mothers received the available up-to-date counselling and information in the area of infant feeding [unpublished data]. Nevertheless, it seems that HIV-positive mothers gravitate towards the worst feeding options in some of the aforementioned respects. We cannot assess how infant feeding counselling affected infant feeding practices, but the message that HIV can be transmitted through breast milk might have produced unwanted consequences. Our view is that replacement feeding should be promoted with great caution, if at all in settings where the WHO's criteria for exclusive replacement feeding is missing: acceptability, feasibility, affordability, sustainability and safety [30,34]. Exclusive breastfeeding needs to be promoted for all infants below the age of 6 months and efforts should be made to reach the least educated and poorest groups. HIV-positive mothers needs information about the importance of avoiding mixed feeding [34]. Earlier identification of HIV with diagnosis prior to birth will also make it possible to take better preventive measures [35].

A comparison of two cross-sectional studies utilising analogous questionnaires in the same study setting at two nearby points in time raises certain methodological challenges. Within the time period of one year there might have been minor changes in behaviour. As there were no major changes in the feeding recommendations from WHO or national guidelines introduced in this period, we believe that the time interval did not affect the observed differences noteworthy. Our cross-sectional design also left out deceased children. From an epidemiological point of view, a case-control design could have been chosen. Pooling of data is nevertheless a cost-reducing, widely-used method, and reliable when used with caution [36]. The questionnaires were not identical, though very similar. It is well documented that 24 hour recall overestimates the prevalence of exclusive breastfeeding [37]. Bland et al. suggested the use of recurrent one-week frequency recalls for better estimation [37]. In the group of HIV-positive mothers, recall periods of one week and 24 hours were compared and gave similar results [29]. Our study may also have been influenced by socially desirable responses. Mothers who were recommended to practise certain feeding options may wish to report those practices. We did not measure this potential bias, but utilising data collectors who were not currently client counsellors probably reduced the threat. The fact that a higher proportion of urban mothers were recruited into the general-population survey may have created a somewhat unbalanced comparison. In fact, urban mothers were more educated than those living in rural areas. Using linear regression to control for living area showed that HIV-positive status was still associated with lower education. Mothers' socio-economic status was similarly associated with HIV status. The general-population mothers were likely to include some HIV-positive cases. Consequently, differences in infant feeding practices between HIV-positive and HIV-negative mothers are likely to be slightly greater than we have reported between the HIV-positive mothers and the general population. These limitations taken into account, we believe the results presented are trustworthy.

## Conclusion

In many respects, HIV-positive mothers fed their infants less favourably than mothers in the general population, with potentially detrimental effects both on the child's nutrition and on the risk of HIV transmission. Mixed feeding and pre-lacteal feeding were widespread in both groups of mothers. The HIV-positive mothers seem to have adapted the duration of breastfeeding to their status, with a shorter duration than the general population.

Infant feeding practices were associated with both mothers' education and socio-economic wealth. Higher educational level and being socio-economically better-off were associated with more beneficial feeding practices.

## Abbreviations

ANOVA: analysis of variance; C.I.: confidence interval; DHS: Demographical health survey; HIV: human immunodeficiency virus; IQR: inter-quartile range; PMTCT: Prevention of Mother-to-Child Transmission; SPSS: Statistical Package for the Social Sciences; TASO: The Aids Support Organisation; WHO: World Health Organisation.

## Competing interests

The authors declare that they have no competing interests.

## Authors' contributions

LTF design, implementation, analysis and writing. IMSE design, implementation, analysis and writing. HW analysis and co-writing. NS implementation of the study and co-writing. TT initiation of the study, design, analysis and co-writing. JKT initiation of the study and co-writing. All authors read and approved the final manuscript.

## Pre-publication history

The pre-publication history for this paper can be accessed here:


